# Elderly Men with De Novo Metastatic Castration-Sensitive Prostate Cancer: Therapy and Does Comorbidity Matter

**DOI:** 10.3390/medicina61112068

**Published:** 2025-11-20

**Authors:** Ugur Ozberk, Selin Akturk Esen, Alican Uguz, Efnan Algın, Oznur Bal, Bulent Akıncı, Dogan Uncu

**Affiliations:** Department of Medical Oncology, Ankara City Hospital, 06800 Ankara, Türkiye; drselin16@hotmail.com (S.A.E.); alicanuguz1@hotmail.com (A.U.); efnanalgin@gmail.com (E.A.); dr_ozn@yahoo.com (O.B.); mbakinci@gmail.com (B.A.); doganuncu@yahoo.com (D.U.)

**Keywords:** prostatic neoplasms, castration-sensitive prostate cancer, elderly patients, treatment outcome

## Abstract

*Background and Objectives*: Prostate cancer (PC) is largely a disease of elderly men, and de novo metastatic presentations are increasingly reported in this population. Yet older patients remain underrepresented in clinical trials, limiting the applicability of guideline-based treatments. Materials and Methods: We retrospectively analyzed 90 patients aged ≥75 years with de novo metastatic castration-sensitive PC (mCSPC) to describe clinical features, treatment patterns, and survival outcomes. *Results*: Median age was 81 years; high-volume disease and Gleason grade 9–10 tumors predominated. A substantial portion of patients received androgen deprivation therapy (ADT) alone or with bicalutamide despite recommendations for intensified therapy. Enzalutamide was associated with the longest median progression-free survival (PFS, 25.4 months) and overall survival (OS, 30.5 months), though between-group differences were not significant. Castration resistance occurred only in the high-volume group (22.4%). Absence of hypertension was associated with a lower risk of progression (HR 0.46, 95% CI 0.23–0.92, *p* = 0.028). *Conclusions*: Elderly patients with de novo mCSPC often have aggressive forms of the disease. Enzalutamide was associated with numerically longer survival compared to other treatments, although the difference was not statistically significant. Additionally, the absence of hypertension appeared to be linked with a lower risk of progression, suggesting that comorbid conditions such as hypertension may influence treatment outcomes in elderly patients.

## 1. Introduction

Prostate cancer (PC) is predominantly a disease of elderly men, with incidence rates increasing substantially with age. While most PC cases are diagnosed at a clinically localized stage [1], there has been a significant rise in the frequency of metastatic presentations in recent years. An analysis of Surveillance, Epidemiology, and End Results (SEER) data from 2004 to 2018 demonstrated that among men aged 75 years and older the incidence of metastatic PC initially declined between 2004 and 2011, but subsequently increased significantly, reaching 88.97 per 100,000 by 2018 [2].

Since the pioneering work of Charles Huggins, who first demonstrated the role of androgen deprivation in the treatment of PC, hormonal therapy has remained a cornerstone in the management of metastatic disease [3,4]. A subset of these patients are classified as having metastatic castration-sensitive PC (mCSPC), which refers to the presence of metastatic disease that still responds to androgen deprivation therapy (ADT). Initially, treatment consisted primarily of ADT alone; however, the therapeutic landscape has evolved significantly with the introduction of novel agents such as androgen receptor pathway inhibitors (e.g., abiraterone, enzalutamide) and chemotherapy agents like docetaxel. These treatment combinations have demonstrated improved overall survival (OS) and are now considered standard of care in appropriately selected patients.

However, despite the emergence of multiple novel treatment options, there is still no consensus on the optimal sequencing of therapies, contributing to uncertainty in the management of this patient population. Furthermore, older patients are often underrepresented in clinical trials [5,6], raising concerns about whether they are receiving the most appropriate treatment. This study aims to contribute to the current understanding of PC management by evaluating cases of de novo mCSPC in elderly men.

## 2. Methods

This retrospective study was conducted at Ankara Bilkent City Hospital, located in Ankara, Turkey. Patients who were over 75 years of age at the time of diagnosis with de novo mCSPC were included in the study. The study cohort comprised patients diagnosed with de novo mCSPC who initiated first-line therapy between 1 January 2018 and 31 December 2024.

Demographic and clinical data, including age, comorbidities, presenting symptoms, prostate-specific antigen (PSA) level at diagnosis, Gleason score, disease volume according to the CHAARTED study [7], risk score based on the LATITUDE study [8], treatments received, and response to these treatments, were collected from hospital records. Radiological assessment was based on findings from gallium-68 prostate-specific membrane antigen positron emission tomography/computed tomography (Ga-68 PSMA PET/CT), conventional computed tomography (CT), or bone scintigraphy performed at baseline. The treatments received by the patients were recorded as docetaxel, abiraterone, enzalutamide, ADT monotherapy, ADT plus bicalutamide, lutetium, or best supportive care. In patients receiving ADT plus bicalutamide, the antiandrogen was administered as part of continuous combined androgen blockade rather than for flare prevention. The choice of first-line treatment (docetaxel, abiraterone, enzalutamide, or ADT ± bicalutamide) was made at the discretion of the treating physician based on patients’ performance status, comorbidities, and overall clinical condition, in accordance with the contemporary clinical guidelines applicable at the time of treatment initiation.

Presenting symptoms were categorized based on clinical documentation at the time of diagnosis. Lower urinary tract symptoms such as frequency, urgency, nocturia, hesitancy, weak stream, or incomplete emptying were considered as one symptom category. Disease volume was defined according to the CHAARTED study criteria [7]. High-volume disease was characterized by the presence of visceral metastases and/or four or more bone lesions, with at least one lesion located beyond the vertebral bodies and pelvis; low-volume disease was defined as having fewer than four bone lesions without visceral involvement. Risk classification was determined based on the LATITUDE study criteria [8]. High-risk disease was defined by the presence of at least two of the following three factors: a Gleason score of 8 or higher, the presence of three or more bone lesions, and the presence of measurable visceral metastases. Patients not meeting these criteria were classified as having low-risk disease.

All patients received continuous ADT using LHRH agonists throughout the treatment period. Castration resistance was defined as disease progression despite castrate levels of serum testosterone (<50 ng/dL or <1.7 nmol/L), as evidenced by a rising PSA level or radiologic progression on Ga-68 PSMA PET/CT, CT, or bone scintigraphy. Progressive disease was defined as radiologic progression or a consecutive rise in PSA levels.

Progression-free survival (PFS) was defined as the time from diagnosis to the first documented evidence of disease progression or death from any cause, whichever occurred first. Overall survival was defined as the time from diagnosis to death from any cause. Patients who were alive without progression at the time of the final follow-up were censored at that date.

The study protocol was approved by the Ethics Committee of Ankara Bilkent City Hospital (Decision No: TABED 1/1095/2025, Date: 12 March 2025), and the study was conducted in accordance with the principles of the Declaration of Helsinki.

### Statistical Analyses

Statistical analyses were performed via IBM SPSS Statistics for Windows Version 25.0 (Statistical Package for the Social Sciences, IBM Corp., Armonk, NY, USA). Descriptive statistics are presented as frequencies and percentages for categorical variables, and medians (minimum–maximum) for continuous variables. When the study data were examined in terms of normality assumptions, Kolmogorov–Smirnov values were determined as *p* < 0.05. Survival analyses were performed using the Kaplan–Meier method, and survival curves were compared using the log-rank test. Univariate Cox proportional hazards regression analyses were conducted to evaluate the association between clinical variables and survival outcomes (PFS and OS). The proportional hazards assumption was evaluated using log-minus-log survival plots and time-dependent covariates; no significant violations were detected. Hazard ratios (HRs) and 95% confidence intervals (CIs) were reported for each variable. Statistical significance was set at *p* < 0.05.

## 3. Results

Table 1 shows the demographic and clinical features of the patients. The median age at diagnosis was 81 (75–93) years. The most common comorbidities were hypertension in 74 patients (82.2%), dyslipidemia in 57 (63.3%), diabetes mellitus in 29 (32.2%), coronary artery disease in 25 (27.8%), and chronic obstructive pulmonary disease (COPD) in 20 (22.2%). Regarding presenting symptoms, bone pain was the most frequent symptom observed in 51 patients (56.7%), followed by lower urinary tract symptoms in 34 (37.7%). Weight loss was reported by only one patient (1.1%), and four patients (4.4%) were asymptomatic, with elevated PSA levels detected during routine screening. The median PSA level at diagnosis was 151 ng/mL (range: 3–4850 ng/mL). At diagnosis, the Gleason grade was 7 in seven patients (7.7%), 8 in 28 (31.1%), and 9–10 in 55 (61.1%). Median follow-up time was 22.3 months.

According to the LATITUDE risk classification, four patients (80%) in the low-volume group were categorized as low-risk, while only two patients (2.4%) in the high-volume group fell into this category. Conversely, high-risk disease was observed in 1 patient (20%) in the low-volume group and in 83 patients (97.6%) in the high-volume group. Among patients with mCSPC, first-line treatment varied between groups. The treatments received by the patients are summarized in Table 2.

Castration resistance developed in 19 patients (22.4%) in the high-volume group following first-line therapy, while no cases of resistance were observed in the low-volume group. All five patients (100%) in the low-volume group remained castration-sensitive, compared to 66 patients (77.6%) in the high-volume group. Among patients who developed castration resistance, the median time to CRPC was 16.2 months (range: 3.0–26.2 months). Of these 19 patients, nine were receiving docetaxel, four were on abiraterone, three on enzalutamide, two on LHRH agonist monotherapy, and one on LHRH agonist plus bicalutamide. The median time to CRPC was 14.0 months for docetaxel, 11.5 months for abiraterone, 17.1 months for enzalutamide, and 10.2 months for LHRH agonist monotherapy. No statistical comparison was performed because of the small subgroup sizes. Regarding first-line treatment in the metastatic castration-resistant PC (mCRPC) setting, five patients (26.3%) received docetaxel, eight (42.1%) received abiraterone, three (15.8%) received enzalutamide, and three (15.8%) received best supportive care. Progression after first-line mCRPC treatment was observed in six patients (31.6%), whereas 13 patients (68.4%) did not show disease progression. Among patients who received second-line treatment for mCRPC, two (33.3%) were treated with lutetium therapy and four (66.7%) received best supportive care (Table 2).

**Figure 1:**
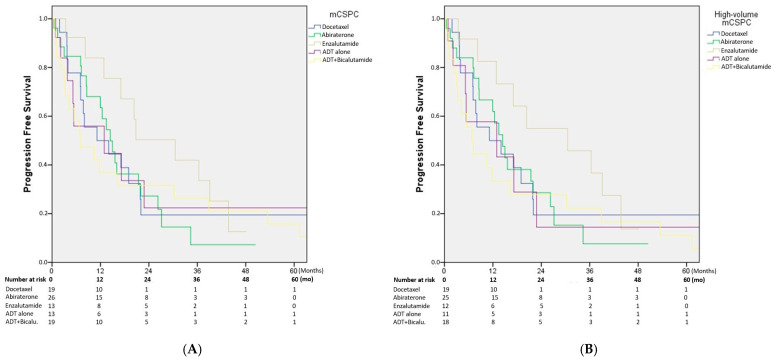
(**A**) Progression-free survival (PFS) for the entire cohort according to first-line treatment (docetaxel, abiraterone, enzalutamide, ADT alone, or ADT plus bicalutamide). Although enzalutamide showed numerically longer PFS compared to other treatments, the difference was not statistically significant (*p* = 0.788). (**B**) PFS in patients with high-volume mCSPC after excluding five patients with low-volume disease. Enzalutamide again demonstrated numerically longer PFS, but the difference was not statistically significant (*p* = 0.665). ADT: androgen deprivation therapy; Bicalu. bicalutamide; mCSPC: metastatic castration-sensitive prostate cancer; Mo: Months.

The median PFS for the entire cohort was 14.4 months (10.8–18.0). In subgroup analyses, the median PFS was 11.1 months (0.1–23.6) for patients treated with docetaxel, 14.4 months (10.8–17.9) for those receiving abiraterone, and 25.4 months (13.6–47.42) for those treated with enzalutamide. In patients who received LHRH agonist alone, the median PFS was 12.9 months (0.1–33.5), whereas it was 7.1 months (0.1–14.2) in those treated with LHRH agonist combined with bicalutamide. Although enzalutamide was associated with a numerically longer PFS compared to the other treatment groups, this difference did not reach statistical significance (*p* = 0.788) (Figure 1A).

After excluding the five patients with low-volume disease, we reanalyzed the data focusing solely on high-volume mCSPC cases. The median PFS for the entire high-volume cohort was 14 months (10.2–17.7). In subgroup analyses, the median PFS was 11.1 months (0.1–23.6) for patients treated with docetaxel, 14.4 months (10.6–18.1) for abiraterone, 30.5 months (4.8–56.2) for enzalutamide, 12.9 months (0.1–30.9) for LHRH agonist alone, and 6.7 months (3.3–10.1) for LHRH agonist combined with bicalutamide. Although enzalutamide showed a numerically longer PFS, this difference did not reach statistical significance (*p* = 0.665) (Figure 1B).

**Figure 2:**
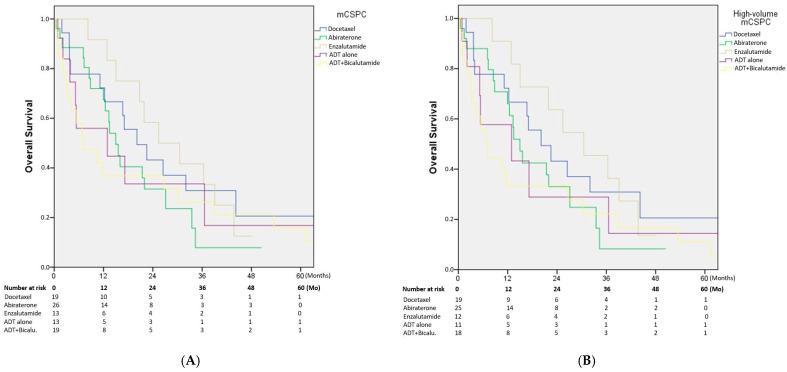
(**A**) Overall survival (OS) for the entire cohort stratified by first-line treatment. Numerically longer OS was observed with enzalutamide and docetaxel; however, the differences were not statistically significant (*p* = 0.562). (**B**) OS in the high-volume mCSPC subgroup after excluding patients with low-volume disease. Enzalutamide and docetaxel were associated with numerically longer survival, but without statistical significance (*p* = 0.562). At baseline, 90 patients were included in the PFS and OS analyses (Figure 1A and Figure 2A). After excluding five low-volume cases, 85 patients remained in the high-volume subgroup analyses (Figure 1B and Figure 2B). The number of patients at risk decreased to approximately 70 at 12 months, about one-third of the cohort at 24 months, and fewer than 10 beyond 36 months in each treatment group. ADT: androgen deprivation therapy; Bicalu. bicalutamide; mCSPC: metastatic castration-sensitive prostate cancer; Mo: Months.

Overall survival in patients with mCSPC was 17 months (10.9–23.1) for the entire cohort. In subgroup analysis, median OS was 20.1 months (9.7–30.5) in patients receiving docetaxel, 14.9 months (11.5–18.4) with abiraterone, 30.5 months (10.7–40.0) with enzalutamide, 12.9 months (0.1–33.5) with LHRH agonist monotherapy, and 7.1 months (0.1–14.2) in those treated with LHRH agonist plus bicalutamide (*p* = 0.562). Although numerically longer survival was observed with enzalutamide and docetaxel, the differences did not reach statistical significance (Figure 2A). 

After excluding the five patients with low-volume disease, the analysis was repeated in the high-volume mCSPC subgroup. In this cohort, median OS was 16.6 months (9.2–24.1). Subgroup analysis showed median OS of 20.1 months (9.7–30.5) with docetaxel, 14.9 months (11.5–18.3) with abiraterone, 30.5 months (14.8–46.1) with enzalutamide, 12.9 months (0.1–30.9) with LHRH agonist monotherapy, and 6.7 months (3.3–10.1) with LHRH agonist plus bicalutamide (*p* = 0.562). Numerically longer survival was observed in patients treated with enzalutamide and docetaxel; however, the differences did not reach statistical significance (Figure 2B).

The most commonly observed side effects were fatigue, hypertension, and hepatic enzyme elevation, which were generally mild to moderate in severity (Grade 1–2) and managed with supportive care or temporary treatment interruption. No treatment-related discontinuations or deaths were recorded.

In univariate Cox regression analysis for PFS, the absence of hypertension was significantly associated with a lower risk of progression (HR: 0.46, 95% CI: 0.23–0.92, *p* = 0.028). Other clinical factors, including age at diagnosis, dyslipidemia, diabetes, COPD, coronary artery disease, PSA at diagnosis, Gleason grade, CHAARTED volume, LATITUDE risk score, and the type of first-line treatment for mCSPC, were not significantly associated with PFS (Table 3).

Univariate Cox regression analysis revealed no statistically significant association between OS and any of the evaluated variables, including age, PSA level, comorbidities, Gleason grade, disease volume and risk classification, type of first-line treatment, or castration resistance (Table 4).

## 4. Discussion

Our study revealed key observations in elderly patients with de novo mCSPC. Among first-line treatment options, enzalutamide was associated with numerically longer PFS and OS compared to other agents, including docetaxel and abiraterone; however, these differences did not reach statistical significance. Similar patterns were observed in both the overall cohort and the high-volume subgroup. Given the small subgroup sizes, these findings should be interpreted cautiously, as the numerical differences may reflect sample variability rather than true efficacy differences. Nonetheless, the consistent direction of the estimates across subgroups may warrant further evaluation in larger studies.

The absence of hypertension emerged as a potential protective factor for disease progression, suggesting that hypertensive patients may experience a more aggressive disease course or reduced treatment efficacy. Increased blood pressure has been linked to steroid hormone–dependent tumors such as PC [9]. Moreover, in several studies, it has been shown that hypertension is associated with an increased risk of mortality from cancer [10,11]. Several mediators, such as nitric oxide (NO) and the renin-angiotensin system may be responsible for this association [12,13,14]. Additionally, some antihypertensive medications may promote cancer [15]. Moreover, different classes of antihypertensive drugs appear to exert divergent effects on prostate cancer outcomes. Renin–angiotensin system (RAS) inhibitors, such as ACE inhibitors and ARBs, have been suggested to improve prognosis by modulating tumor microcirculation and suppressing proliferative signaling [16]. Conversely, some studies have reported that diuretic use may be linked to an increased risk of prostate cancer mortality [17].Although the exact underlying mechanisms remain unclear, this finding underscores the importance of incorporating comorbid conditions such as hypertension into the prognostic assessment and therapeutic planning for patients with mCSPC.

The predominance of Gleason grade 9–10 and high-volume disease in our cohort suggests that de novo mCSPC in elderly patients often presents with aggressive histopathology and a substantial metastatic burden. This finding is consistent with previous literature, which indicates that the incidence of high-grade tumors (Gleason score 8–10) increases with age [18]. Additionally, it is well established that older patients are more likely to be diagnosed at advanced stages compared to their younger counterparts [19]. Several factors may contribute to this observation: tumors may exhibit more rapid growth in older individuals [20]; less frequent PSA testing and reduced use of advanced diagnostic evaluations may lead to delayed detection [21]; due to considerations related to limited life expectancy, coexisting medical conditions, the burden of multiple medications, economic challenges, and social issues influencing caregiving responsibilities [22].

In our cohort, a substantial proportion of patients with mCSPC received either ADT alone or in combination with bicalutamide. Although current guidelines do not recommend ADT or ADT plus bicalutamide as standard treatment options for mCSPC [23], the frequent use of these regimens, particularly among elderly patients, highlights the challenges in adhering to guideline-recommended therapies in this population. This may, in turn, contribute to increased mortality rates in patients who do not receive optimal treatment.

In our cohort, castration resistance predominantly developed in patients within the high-volume disease group. This may suggest that the emergence of castration resistance is more closely related to the aggressive biological behavior of the disease rather than simply the tumor burden. Among those who developed castration resistance, approximately one-third experienced further disease progression despite treatment. The majority of patients received best supportive care (BSC) rather than second-line therapy, which may reflect the influence of advanced age, comorbid conditions, and the extent of disease progression.

This study has several limitations that should be acknowledged. First, its retrospective design and single-center nature may limit the generalizability of the findings. Second, the relatively small sample size, especially within the treatment subgroups, reduced the statistical power to detect significant differences, particularly in survival outcomes. Third, the absence of molecular or genomic profiling restricts the ability to evaluate underlying biological mechanisms contributing to disease aggressiveness or treatment resistance in this elderly population. Finally, given the limited number of patients and events, a formal competing-risks analysis was not feasible, which may represent a methodological limitation in this elderly cohort.

## 5. Conclusions

This study highlights key clinical characteristics and treatment outcomes in elderly patients with de novo mCSPC. The predominance of high-grade, high-volume disease underscores the aggressive nature of PC in this age group. Despite guideline recommendations, a substantial proportion of patients received ADT alone or in combination with bicalutamide, reflecting real-world challenges in applying intensified regimens to geriatric populations. Enzalutamide was associated with numerically longer PFS and OS, including among those with high-volume disease, although these differences were not statistically significant. These findings should be interpreted with caution given the small sample size, yet the consistent direction of the estimates suggests that further investigation of enzalutamide in elderly patients may be warranted. The observed association between hypertension and disease progression also merits additional research into the role of comorbid conditions in prostate cancer outcomes.

## Figures and Tables

**Table 1:** Demographic and Clinical Features of The Patients.

**Age at Diagnosis (Year)**	**81 (75–93)**
**Comorbidities** (n, %)	
Hypertension	74 (82.2)
Dyslipidemia	57 (63.3)
Diabetes mellitus	29 (32.2)
Chronic obstructive pulmonary disease	20 (22.2)
Coronary artery disease	25 (27.8)
**Presenting symptom (n, %)**	
Lower urinary tract symptoms	34 (37.7)
Bone pain	51 (56.7)
Weight loss	1 (1.1)
Asymptomatic (PSA elevation on screening)	4 (4.4)
**PSA level at diagnosis (interquartile range) (** **ng/mL)**	151 (3–4850)
**Gleason grade at diagnosis (n, %)**	
Grade 7	7 (7.7)
Grade 8	28 (31.1)
Grade 9–10	55 (61.1)
**Median follow-up (interquartile range) (months)**	22.3 (11.4–34.7)

**Table 2:** Disease Volume-Based Distribution of Risk Classification, Treatment, and Progression in mCSPC.

	Low Volume (n, %)	High Volume (n, %)
**Risk category (LATITUDE)**		
Low risk	4 (80)	2 (2.4)
High risk	1 (20)	83 (97.6)
**First-line treatment in mCSPC**		
Docetaxel	–	19 (22.4)
Abiraterone	1 (20)	25 (29.4)
Enzalutamide	1 (20)	12 (14.1)
ADT monotherapy	2 (40)	11 (12.9)
ADT + bicalutamide	1 (20)	18 (21.2)
**Castration resistance following first-line therapy**		
Yes	–	19 (22.4)
No	5 (100)	66 (77.6)
**First-line treatment in mCRPC**		
Docetaxel	–	5 (26.3)
Abiraterone	–	8 (42.1)
Enzalutamide	–	3 (15.8)
Best supportive care	–	3 (15.8)
**Progression after first-line treatment in mCRPC**		
Yes	–	6 (31.6)
No	–	13 (68.4)
**Second-line treatment in mCRPC**		
Lutetium	–	2 (33.3)
Best supportive care	–	4 (66.7)

**Table 3:** Univariate Cox Regression Analysis for Progression-Free Survival (PFS).

Variable		HR (95% CI)	*p*-Value
**Age at diagnosis**	–	1.05 (0.99–1.11)	0.056
**Hypertension**	Yes (ref)	1.00	–
	No	0.46 (0.23–0.92)	**0.028**
**Dyslipidemia**	Yes (ref)	1.00	–
	No	0.91 (0.55–1.50)	0.734
**Diabetes**	Yes (ref)	1.00	–
	No	0.86 (0.52–1.42)	0.568
**COPD**	Yes (ref)	1.00	–
	No	1.12 (0.63–2.00)	0.677
**Coronary artery disease (CAD)**	Yes (ref)	1.00	–
	No	1.23 (0.72–2.09)	0.435
**PSA at diagnosis** (per 10 ng/mL increase)	–	1.000 (0.997–1.003)	0.656
**Gleason grade**	Grade 7 (ref)	1.00	–
	Grade 8	0.83 (0.28–2.50)	0.752
	Grade 9–10	1.12 (0.40–3.15)	0.752
**CHAARTED volume**	Low-volume (ref)	1.00	–
	High-volume	1.43 (0.51–3.94)	0.487
**LATITUDE risk**	Low-risk (ref)	1.00	–
	High-risk	1.39 (0.56–3.48)	0.474
**First-line treatment in mCSPC**	Docetaxel (ref)	1.00	–
	Abiraterone	1.05 (0.53–2.06)	0.793
	Enzalutamide	0.66 (0.29–1.49)	0.793
	LHRH agonist monotherapy	0.99 (0.42–2.28)	0.793
	LHRH agonist + Bicalutamide	1.06 (0.53–2.13)	0.793

**Table 4:** Univariate Cox Regression Analysis for Overall Survival (OS).

Variable		HR (95% CI)	*p*-Value
**Age at diagnosis**		0.97 (0.85–1.10)	0.649
**Hypertension**	Yes (ref)	1.00	–
	No	0.52 (0.16–1.67)	0.275
**Dyslipidemia**	Yes (ref)	1.00	–
	No	1.36 (0.54–3.39)	0.510
**Diabetes**	Yes (ref)	1.00	–
	No	1.04 (0.37–2.93)	0.934
**COPD**	Yes (ref)	1.00	–
	No	0.61 (0.23–1.63)	0.332
**Coronary artery disease**	Yes (ref)	1.00	–
	No	0.87 (0.33–2.33)	0.794
**PSA at diagnosis** (per 10 ng/mL increase)		1.000 (0.997–1.003)	0.260
**Gleason grade**	Grade 7 (ref)	1.00	–
	Grade 8	3.38 (0.43–26.50)	0.088
	Grade 9–10	1.25 (0.15–10.11)	0.088
**CHAARTED volume**	Low-volume (ref)	1.00	–
	High-volume	1.49 (0.19–11.20)	0.698
**LATITUDE risk**	Low-risk (ref)	1.00	–
	High-risk	1.90 (0.25–14.30)	0.533
**First-line treatment in mCSPC**	Docetaxel (ref)	1.00	–
	Abiraterone	1.75 (0.53–5.76)	0.776
	Enzalutamide	0.84 (0.19–3.62)	0.776
	LHRH agonist monotherapy	1.72 (0.46–6.43)	0.776
	LHRH agonist + Bicalutamide	1.98 (0.69–5.49)	0.776
**Castration resistance**	No (ref)	1.00	–
	Yes	0.28 (0.06–1.22)	0.092

## Data Availability

Due to ethical and privacy restrictions, the raw data are not publicly available. The datasets generated and analyzed during the current study are available from the corresponding author on reasonable request.
